# Enhanced Message-Passing Based LEACH Protocol for Wireless Sensor Networks

**DOI:** 10.3390/s19010075

**Published:** 2018-12-25

**Authors:** Jaeyoung Kang, Illsoo Sohn, Sang Hyun Lee

**Affiliations:** 1 School of Electrical Engineering, Korea University, Seoul 02841, Korea; retbird13@korea.ac.kr; 2 Department of Computer Science and Engineering, Seoul National University of Science and Technology, Seoul 01811, Korea; isohn@seoultech.ac.kr

**Keywords:** LEACH, message passing, nonlinear power consumption

## Abstract

This paper proposes a distributed energy-efficient clustering protocol for wireless sensor networks (WSNs). Based on low-energy adaptive clustering hierarchy (LEACH) protocol, the proposed LEACH-eXtended Message-Passing (LEACH-XMP) substantially improves a cluster formation algorithm, which is critical for WSN operations. Unlike the previous approaches, a realistic non-linear energy consumption model is considered, which renders the clustering optimization highly nonlinear and challenging. To this end, a state-of-the-art message-passing approach is introduced to develop an efficient distributed algorithm. The main benefits of the proposed technique are its inherent nature of a distributed algorithm and the saving of computational load imposed for each node. Thus, it proves useful for a practical deployment. In addition, the proposed algorithm rapidly converges to a very accurate solution within a few iterations. Simulation results ensure that the proposed LEACH-XMP maximizes the network lifetime and outperforms existing techniques consistently.

## 1. Introduction

Advances in sensor technology popularize battery-powered wireless sensor networks (WSNs) in a large number of industrial areas including vehicle traffic monitoring, smart factories, internet-of-things devices, and public safety networks to name a few [1,2]. As sophisticated functional features are necessary to achieve a high level of the automation for conveniences, additional wireless sensors are accommodated for covering an increasing demand of the sensing data. On top of a sophisticated network formation, sensors are subject to a variety of physical environments where the cost of intervening in the sensor network is massive [3,4]. Therefore, it is indispensable to extend the lifetime of the WSN.

There have been a number of efforts to maximize the longevity of the WSN, such as mobile relays, optimal deployment of sensor nodes, and energy harvesting [5]. In terms of routing and clustering, low-energy adaptive clustering hierarchy (LEACH) protocol has played a trailblazing role in the minimization of the energy consumption in the network [6]. This protocol uses a hierarchical clustering-based routing strategy which forms multiple clusters of nodes, each with a single cluster head (CH) node. In each group, member nodes transfer their information to the CH node, and the CH node is responsible for aggregating whole received information and forwarding it to the base station (BS). Besides single-hop clustering, there are several multi-hop hierarchical clustering based protocol. Power-efficient gathering in sensor information systems (PEGASIS) forms chains of sensor nodes such that each node transfers the data back and forth with adjacent nodes [7]. Hybrid energy-efficient distributed clustering (HEED) considers the residual energy and the proximity to adjacent nodes for the selection of the CH node [8]. In addition, energy-efficient uneven clustering (EEUC) protocol partitions the set of sensor nodes into unequal sized clusters and uses multi-hop routing for inter-cluster communication to save the energy of the CH node located near the BS [9].

For simplicity of the LEACH protocol, several variations have been developed for the minimization of the network energy consumption. In LEACH-C [6], a central coordinator exists to control the cluster formation. By collecting all required network information, the central coordinator provides a solution for the optimized clusters. In LEACH-CE [10], a cluster head selection has been improved. Upon the formation of a cluster, the member node with the highest remaining energy is selected as a CH node. This enables to distribute the energy consumption load uniformly over the member nodes. In LEACH-CKM [11], a *K*-means (or *K*-medians) clustering algorithm has been adopted to enhance the clustering performance. With a sophisticated energy-efficient cluster formation, the WSN extends its lifetime.

However, aforementioned centralized protocols impose several practical requirements. They require accurate location information of all sensor nodes in the network. Additional hardware with localizing features, e.g., GPS, result in an increase in the cost and power consumption for sensor devices. Furthermore, the collection of the location information of individual nodes and the distribution of the clustering decision by the network coordinator become a signaling burden to the network. To cope with the practical issues, LEACH-AP has been proposed as a distributed solution [12]. LEACH-AP develops a distributed algorithm based on affinity propagation [13]. Hence, the location information of sensor nodes is not necessary and the signaling overhead becomes minimal. In addition, it outperforms the existing centralized counterparts in terms of the network lifetime. However, there are some drawbacks of LEACH-AP. LEACH-AP simplifies an energy consumption model of CH nodes for mathematical tractability [12]. The assumption that the sensing area is apart away from the BS and transmission energy dominates the energy consumption of CH nodes ignores the impact of the dynamic cluster size on the energy consumption of CH nodes in the simplified model. Thus, variable energy consumption for CH nodes has not been properly included, thereby resulting in an occasional deviation from the guaranteed optimality. In addition, LEACH-AP assumes that the sensing data within a cluster is closely correlated and thus all sensing data collected at the CH node are perfectly compressed. This assumption means that the size of the aggregated data at CH nodes is the same as the sensing data of each sensor node. In reality, the correlation between the sensing data of neighboring sensor nodes varies. The size of the aggregated data at CH nodes also differs depending on the number of data and their correlation, and may degrade the performance of the simplified model. However, revising the simplified energy consumption model of CH nodes to reflect a new feature renders the model highly nonlinear.

To resolve the issue, this work develops an energy consumption-minimizing strategy called LEACH-XMP (LEACH-eXtended Message-Passing) based on an enhanced technique of the message- passing algorithm [14]. Unlike the previous approach in [12], the LEACH-XMP adopts a sophisticated but realistic nonlinear energy consumption model for CH nodes without oversimplification. The resulting solution is very efficient in improving the cluster formation and in the lifetime maximization of whole WSN. The main contributions of this paper are summarized as follows:

- Generalized energy consumption model: This paper adopts a realistic energy consumption model of a sensor node. The model considers the transmission power dissipation and digital-processing power related to data decoding and aggregation. We capture the impact of the dynamic cluster size on the digital-processing power of a CH node which has been unaddressed in the previous studies. In addition, the data compression rate based on the correlation of the collected data is considered. It influences the transmission power dissipation of a CH node since an inaccurate energy consumption model of CH nodes incurs a suboptimal cluster formation. None of the previous works have properly addressed this issue to the best of the authors’ knowledge.
- Distributed algorithm: The new energy consumption model renders the optimization formulation highly nonlinear. We propose a message-passing approach that can tackle this nonlinearity very efficiently. We construct a graphical model that corresponds to the WSN of interest and develop an efficient distributed algorithm based on it. The advantages of the proposed algorithm include that (i) the algorithm is distributed by nature; (ii) computational load for each node is minimal; and (iii) the algorithm converges rapidly with only a small number of message-passing iteration. The advantages characterize the usefulness of the proposed algorithm for practical implementations.
- Performance: The performance of the proposed algorithm is verified with extensive simulation results. The proposed algorithm outperforms all the previous approaches in terms of energy consumption rate, total delivered data, and network lifetime. In particular, the proposed algorithm improves the network lifetime up to 28.5% compared to existing LEACH protocols in the network lifetime. Based on the realistic energy consumption model, the proposed algorithm provides an efficient solution for different network sizes. As the network size grows, the improvement also increases. This is one of the prominent main benefits of the proposed algorithm.

This paper is organized as follows: Section 2 describes the system model of generalized LEACH and Section 3 specifies the algorithm of LEACH-XMP. Subsequently, Section 4 provides simulation results of proposed algorithm. Finally, Section 5 draws the conclusion.

## 2. System Model

**Figure 1:**
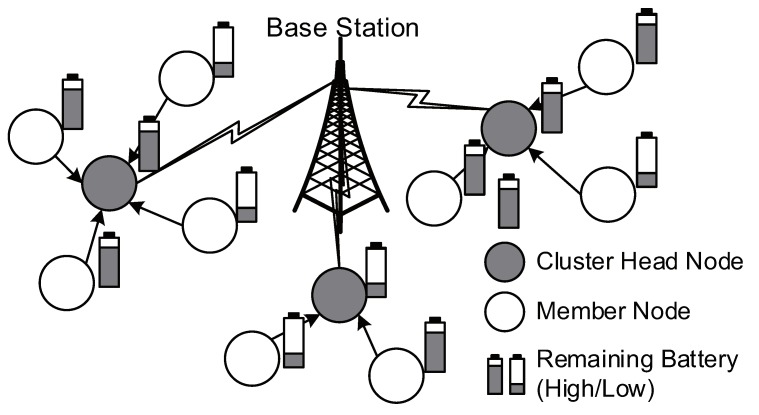
A battery-powered wireless sensor network (WSN) with low-energy adaptive clustering hierarchy (LEACH).

Consider a WSN that deploys a set of *N* sensor nodes denoted by S (see Figure 1). A single BS exists in the network and receives environmental sensing data from sensor nodes. Sensor nodes initially broadcast their identification number and remaining energy information. The received signal strength is used to estimate the distances from neighboring nodes. The LEACH protocol [6] proceeds with set-up phase and steady-state phase. In the set-up phase, clusters are constructed by grouping sensor nodes, where time-slot-based scheduling is also determined within each cluster. In the following steady-state phase, sensing packets from sensor nodes are collected at their CH nodes and then forwarded to the BS.

We desire to maximize the lifetime of the WSN. Such a goal is analogous to maximizing the remaining energy of the WSN. The remaining average total energy of sensors the WSN is monitored to maximize on a regular basis. Let $\tau_{k}$ be the *k*-th time instant where the measurement from sensors is reported to the BS. At time instant $\tau_{k}$, all sensors measure their remaining energy along with the sensing data and share such information with neighbors. If at least one of their neighbors no longer work, they reconfigure the WSN to maximize the remaining energy. We assume that all sensors have the same amount of the initial energy denoted by $E_{0}$. Let $E_{j}^{\left(k\right)}$ and ${\bar{E}}^{\left(k\right)}$ denote the remaining energy of sensor *j* and the average remaining energy of live nodes in the WSN at time instant $\tau_{k}$, respectively. Thus, it holds that $E_{j}^{\left(0\right)}=E_{0}$ for all sensors. Also, the set of live sensors at time instant $\tau_{k}$ is denoted by $S_{k}$. Once the WSN is configured such that all sensors have their own roles in the network, sensor *j* consumes the energy $E_{j}^{\left(k-1\right)}$ depending on its role during the interval $\left[\tau_{k-1},\tau_{k}\right]$. The remaining energy of sensor *j* is given by

(1) $$\begin{array}{c} E_{j}^{\left(k\right)}=E_{j}^{\left(k-1\right)}-E_{j}^{\left(k-1\right)} \end{array}$$

and the the average remaining energy of live sensors is given by

(2) $$\begin{array}{c} {\bar{E}}^{\left(k\right)}={\bar{E}}^{\left(k-1\right)}-\frac{1}{|S_{k}|}\sum_{j\in S_{k}}E_{j}^{\left(k-1\right)} \end{array}$$

Hence, the maximization of the total remaining energy becomes equivalent to the minimization of the energy (corresponding to the second term in (2)) for the communication at each reporting interval.

The energy consumption of a sensor node consists of the transmission energy dissipation and the digital-processing energy consumption proportional to the amount of the processed data [6]. The transmission power dissipation depends on the amount of processed data and the propagation loss. To quantify this, we let $E_{elec}$, $\epsilon_{fs}$, and $\epsilon_{mp}$ denote signal-processing energy per bit, transmission energy per bit in free-space propagation, and the transmission energy per bit in multipath propagation, respectively. The energy consumption of a single sensor varies from the wireless propagation model. According to the energy consumption model in [6], the free-space propagation becomes dominant when the transmission distance is small, while the multipath propagation prevails in the opposite case. A pairwise transmission is assumed to convey *b* bits of sensing data. The energy consumption of node *i* transmitting to node *j* is denote by $w_{ij}$ and is given by

(3) $$w_{ij}=\left\{\begin{array}{cc} bE_{elec}+b\epsilon_{mp}d_{ij}^{4} & \mathrm{if}\;d_{ij}>d_{0},\\ bE_{elec}+b\epsilon_{fs}d_{ij}^{2} & \mathrm{otherwise}, \end{array}\right.$$

where $d_{0}\equiv \sqrt{\epsilon_{fs}/\epsilon_{mp}}$ is the threshold distance associated with the point where the energy consumptions by multipath and free-space propagation are identical. A CH node is responsible for processing and aggregating the received data into a single packet to transfer to the BS. In addition to the energy dissipation of $E_{elec}$ per bit for the signal reception and transmission, a CH node consumes $E_{da}$ per bit to aggregate own received data. For an *n*-member cluster, the CH node transmits the collected information of nb bits to the BS. In previous works [6], it is assumed that the CH node can compress the collected information of nb bits into a single data packet of a constant length *b* to transmit to the BS. However, in practice, the aggregated information may not be compressed into a sufficiently compact data packet. The number of bits required for representing the information can increase according to the relationship among the data from distinct nodes. To characterize it, we consider a generalized model by considering the correlation parameter between different data, denoted by λ. This parameter, taking on a value from [0,1], is, in fact, associated with the best rate which an individual member signal can be compressed with. Let $b_{n}$ denote the number of bits that the CH node of an *n*-member cluster can obtain by compressing *n* different data of *b* bits. Under the assumption of uniform λ among all data from individual sensors, $b_{n}$ is evaluated by the inclusion-exclusion principle as a nonlinear function of *n*, which can be expressed as

(4) $$b_{n}=\frac{1-{\left(1-\lambda \right)}^{n}}{\lambda }b$$

**Figure 2:**
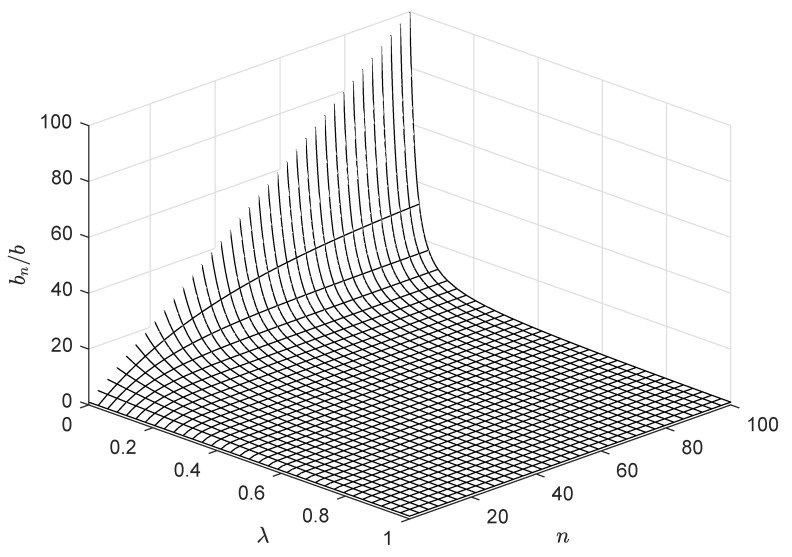
Representation of $b_{n}/b$ for varying λ and *n*.

Note that, if λ is zero, i.e., all measurements are independent of each other and share no common information, $b_{n}$ becomes nb. On the other hand, if λ is one, i.e., all measurements are identical and only one measurement is enough, $b_{n}$ becomes *b*, which corresponds to the assumption made in previous works [6]. This function increases sublinearly with respect to *n* but decreasing with respect to λ as depicted in Figure 2. Thus, the transmitting energy cost per bit of CH node *j* which is apart from the BS by the distance $d_{j}$ amounts to

(5) $$w_{j}=\left\{\begin{array}{cc} E_{elec}+\epsilon_{mp}d_{j}^{4} & \mathrm{if}\;d_{j}>d_{0},\\ E_{elec}+\epsilon_{fs}d_{j}^{2} & \mathrm{otherwise}. \end{array}\right.$$

We include additional requirement for the eligibility of a CH node [12]. Only a node that holds $E_{j}^{\left(k\right)}\geq {\bar{E}}^{\left(k\right)}$ is eligible for the CH selection. Here, ${\bar{E}}^{\left(k\right)}$ can be acquired by consensus-based techniques [15] without global notification of the state of individual nodes.

For clustering formation, several decision tasks are fulfilled such that the number of clusters and their CH nodes are identified and member nodes are affiliated to one of those clusters. Each node decides its own role between CH and member node by considering the corresponding energy consumptions as similarity measures used in the clustering problem. Also, once a node is chosen as a CH node, it cannot become a member node of another cluster. These decisions boil down to two requirements: (i) Each node chooses its CH node. To become a CH node, the node should choose itself as its CH node; (ii) once a node is chosen by other nodes to act as a CH node, it cannot choose another node as its CH node. For concrete mathematical formulation, we introduce binary variable $x_{ij}$ to indicate the membership of node *i*, such that $x_{ij}=1$ indicates node *i* selects node *j* as a CH node. Once node *i* selects node *j* as its CH node, node *j* should choose itself as own CH node, i.e., $x_{jj}=1$. Let N(j) and $u_{j}$ be the set of neighboring nodes around node *j* and the number of neighboring nodes connected to CH node *j* (excluding CH node *j*), respectively. It holds that $u_{j}=\sum_{i\in N(j)}x_{ij}$. We can reformulate the energy consumption of CH node *j* as   

(6) $$\begin{array}{c} f\left(x_{jj},u_{j}\right)=\left\{\begin{array}{cc} 0 & \mathrm{if}\;x_{jj}=0,\\ bw_{j} & \mathrm{else}\;\mathrm{if}\;u_{j}=0,\\ u_{j}bE_{elec}+\left(u_{j}+1\right)bE_{da}+b_{u_{j}+1}w_{j} & \mathrm{else}\;\mathrm{if}\;u_{j}>0. \end{array}\right. \end{array}$$

Note that unlike the simplified model in [12], Equation (6) captures a nonlinear energy consumption model of a CH node. The resulting formulation of the WSN is given by

(7a) $$\begin{array}{cc} \min_{\left\{x_{ij},u_{j}\right\}} & \;\sum_{i\in S_{k}}\sum_{j\in N(i)}w_{ij}x_{ij}+\sum_{j\in S_{k}}f\left(x_{jj},u_{j}\right) \end{array}$$

(7b) $$\begin{array}{cc} \mathrm{subject}\;\mathrm{to} & \;\sum_{j\in N(i)}x_{ij}=1,\;\forall i \end{array}$$

(7c) $$\begin{array}{cc} & \;\sum_{i\in N(j)}x_{ij}=u_{j}x_{jj},\;\forall j \end{array}$$

(7d) $$\begin{array}{cc} & \;x_{ij}\in \left\{0,1\right\},\;u_{j}\in \left\{0,\ldots ,|N\left(j\right)|\right\}, \end{array}$$

where |N(j)| is the number of neighbors around node *j*. The first term of the objective function in (7a) corresponds to the energy consumption of member nodes and the second term corresponds to the energy consumption of CH nodes. The first constraint indicates that each member can select only one CH node and the second constraint dictates that the selected node chooses itself as a CH node of the affiliated cluster. Note that solving the optimization in (7a)–(7d) is a computationally demanding task since the problem is inherently of combinatorial nature in addition to high nonlinearity of objective function and constraints.

## 3. Proposed LEACH-XMP Protocol

### 3.1. Formulation

**Figure 3:**
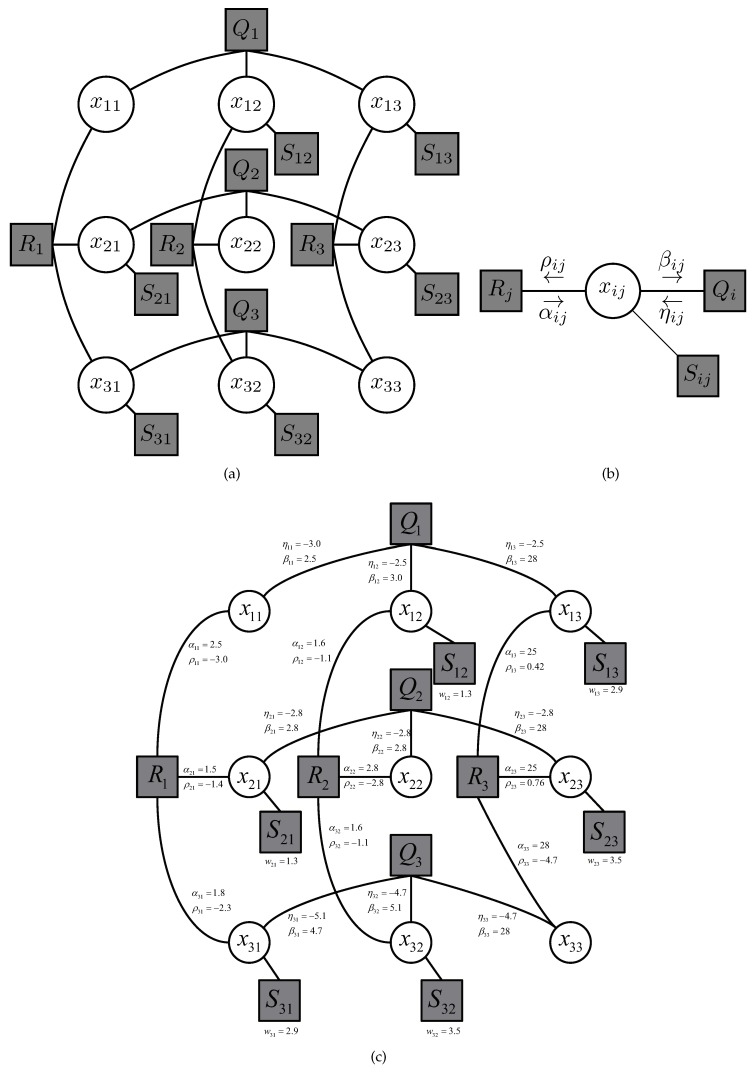
Graphical models. (**a**) Graphical model of 3-node WSN; (**b**) Message definition around a single variable; (**c**) Example of message-passing operation for 3-node WSN.

To tackle the optimization problem in (7a)–(7d), we use a message-passing algorithm which has had remarkable success in various applications [12,13,14]. For the development of a distributed algorithm, Equations (7a)–(7d) is reformulated to an unconstrained optimization as

(8) $$\min_{\left\{x_{ij}\right\}}F\left(x_{ij}\right)\equiv \sum_{i\in S_{k}}\sum_{j\in N(i)}S_{ij}\left(x_{ij}\right)+\sum_{i\in S_{k}}Q_{i}\left(\left\{x_{ij}\right\}\right)+\sum_{j\in S_{k}}R_{j}\left(\left\{x_{ij}\right\}\right)$$

where new functions are defined to enforce the constraints as

(9) $$\begin{array}{cc} S_{ij}\left(x_{ij}\right)= & w_{ij}x_{ij} \end{array}$$

(10) $$\begin{array}{cc} Q_{i}\left(\left\{x_{ij}\right\}\right)= & \left\{\begin{array}{cc} \infty & \mathrm{if}\;\sum_{j\in N(i)}x_{ij}\neq 1\\ 0 & \mathrm{otherwise} \end{array}\right. \end{array}$$

(11) $$\begin{array}{cc} R_{j}\left(\left\{x_{ij}\right\}\right)= & \left\{\begin{array}{cc} \infty & \mathrm{if}\;E_{j}^{\left(k\right)}<{\bar{E}}^{\left(k\right)}\;\mathrm{or}\sum_{i\in N(j)}x_{ij}\neq u_{j}x_{jj}\\ f(x_{jj},u_{j}) & \mathrm{otherwise} \end{array}\right. \end{array}$$

where $S_{ij}\left(x_{ij}\right)$ is introduced to represent the individual contribution of a member node to the total energy consumption of the WSN in (7a), and $Q_{i}\left(\left\{x_{ij}\right\}\right)$ is associated with the constraint (7b) that each sensor node selects its own single CH node. To see this, if node *i* chooses more than two neighboring nodes as its CH node, i.e., there exists more than j∈N(i) such that $x_{ij}=1$, the value of $Q_{i}\left(\left\{x_{ij}\right\}\right)$ becomes infinity, and the objective function in (8) is never minimized. Otherwise, $Q_{i}\left(\left\{x_{ij}\right\}\right)$ takes the value of zero and does not contribute to the final objective function. Thus, the value of (8) becomes identical to the objective of the original problem in (7a)–(7d) when minimized. Also, $R_{j}\left(\left\{x_{ij}\right\}\right)$ represents the constraint for a CH node in (7c) and its energy consumption. If the corresponding constraint is violated, the value of $R_{j}\left(\left\{x_{ij}\right\}\right)$ becomes infinity and the minimization does not work. Otherwise, $R_{j}\left(\left\{x_{ij}\right\}\right)$ takes the value of $f(x_{jj},u_{j})$ which contributes to the total energy consumption of the network. Note here that $R_{j}\left(\left\{x_{ij}\right\}\right)$ is a function of $\left\{x_{ij}\right\}$ solely since $u_{j}$ is thought of as a function of $\left\{x_{ij}\right\}$. In turn, a graphical model associated with (8) is constructed. The graphical model is a bipartite graph consisting of two groups of nodes associated with variables (circles) and functions (boxes) interconnected by the set of edges, each representing the membership of a variable in the corresponding function. Figure 3a shows a graphical model of 3-node WSN. A message-passing algorithm exchanges real-valued quantities called messages along two directions of each edge in the graphical model to obtain a distributed solution. Since there are three different types of function (9)–(11), six types of messages could be defined. However, only a single variable is connected with (9) and the corresponding message remains a trivial constant. Thus, four different messages (as illustrated in Figure 3b) suffice to obtain the message-passing algorithm. A single iteration consists in updating each message along edges between function and variable nodes. This iteration is repeated until all message converge. The converged messages are associated with the preference of the value that each variable can take in the optimal solution i.e., if a message is positive, the corresponding binary variable becomes one in the optimal solution. Figure 3c shows an example of the result of message-passing algorithm upon convergence. The sum of a pair of messages transferred in the opposite directions determines the value of a variable, which is associated with the CH-member node relationship. Note that this example corresponds to the case where node 1 is a CH node of the cluster that contains node 3, while node 2 stands alone.

### 3.2. Message Updates

The message-passing algorithm finds the solution of the unconstrained formulation (8). The algorithm runs in such an iterative fashion that two real-valued messages are transferred between a variable and a factor in two reciprocal directions of the in-between edge. The iterations of the message transfer are repeated until all messages converge to a fixed assignment of variables. Upon convergence of messages, the associated solution has been proved to be optimal in various applications.

Let $\mu_{A\to B}\left(C=c\right)$ denote a message that node *A* transfers node *B* to convey the chance of variable *C* taking the value of *c*. The information contained in the message can be thought of as the preference that optimal solution takes variable *C* equal to *c*. For convenience of representation, four different messages, respectively associated with two directions of edges connected to two factor functions, are reexpressed as:

(12) $$\begin{array}{cc} \beta_{ij}= & \mu_{x_{ij}\to Q_{i}}\left(x_{ij}=1\right)-\mu_{x_{ij}\to Q_{i}}\left(x_{ij}=0\right),\\ \eta_{ij}= & \mu_{Q_{i}\to x_{ij}}\left(x_{ij}=1\right)-\mu_{Q_{i}\to x_{ij}}\left(x_{ij}=0\right),\\ \rho_{ij}= & \mu_{x_{ij}\to R_{i}}\left(x_{ij}=1\right)-\mu_{x_{ij}\to R_{i}}\left(x_{ij}=0\right),\\ \alpha_{ij}= & \mu_{R_{i}\to x_{ij}}\left(x_{ij}=1\right)-\mu_{R_{i}\to x_{ij}}\left(x_{ij}=0\right). \end{array}$$

We first consider messages that sent from variable node to constraint node. According to [14], $\beta_{ij}$ can be calculated by the sum of all incoming messages to the variable node and is given by

(13) $$\begin{array}{c} \beta_{ij}=\left\{\begin{array}{cc} \alpha_{ij}+w_{ij} & \mathrm{if}\;i\neq j,\\ \alpha_{ii} & \mathrm{otherwise}. \end{array}\right. \end{array}$$

Also, $\eta_{ij}$ can also be obtained in the same way as $\beta_{ij}$ and is given by

(14) $$\begin{array}{c} \rho_{ij}=\left\{\begin{array}{cc} \eta_{ij}+w_{ij} & \mathrm{if}\;i\neq j,\\ \eta_{ii} & \mathrm{otherwise}. \end{array}\right. \end{array}$$

The message update rules for messages sent from a constraint node to a variable node are much complicated. The message sent from $x_{ij}$ to function $Q_{i}\left(\left\{x_{ij}\right\}\right)$ is considered first. Note that only one variable associated with function $Q_{i}\left(\left\{x_{ij}\right\}\right)$ can take the value of one. If all incoming messages from other variables are negative, the outgoing message should be positive. By some algebra, $\eta_{ij}$ is obtained in a simple form as   

(15) $$\begin{array}{cc} \eta_{ij}= & \mu_{Q_{i}\to x_{ij}}\left(x_{ij}=1\right)-\mu_{Q_{i}\to x_{ij}}\left(x_{ia}=0\right)\\ \;= & \min_{X_{i}\backslash x_{ij}}Q_{i}\left(x_{ij}=1,X_{i}\backslash x_{ij}\right)+\sum_{k\in N(i)\backslash j}\mu_{x_{ik}\to Q_{i}}\left(x_{ik}\right)-\min_{X_{i}\backslash x_{ij}}Q_{i}\left(x_{ij}=0,X_{i}\backslash x_{ij}\right)+\sum_{k\in N(i)\backslash j}\mu_{x_{ik}\to Q_{i}}\left(x_{ik}\right)\\ \;= & \sum_{k\in N(i)\backslash j}\mu_{x_{ik}\to Q_{i}}\left(0\right)-\min_{k\in N(i)\backslash j}\left(\mu_{x_{ik}\to Q_{i}}\left(1\right)+\sum_{l\notin N(i)\backslash \{j,k\}}\mu_{x_{il}\to Q_{i}}\left(0\right)\right)\\ \;= & -\min_{k\in N(i)\backslash j}\left(\mu_{x_{ik}\to Q_{i}}\left(1\right)-\mu_{x_{ik}\to Q_{i}}\left(0\right)\right)\\ \;= & -\min_{k\in N(i)\backslash j}\beta_{ik}. \end{array}$$

For the sake of simple representation, all three message rules in (13)–(15) are combined to obtain a single message update rule given by

(16) $$\begin{array}{c} \rho_{ij}=w_{ij}-\min_{k\in N(i)\backslash j}\left(w_{ik}+\alpha_{ik}\right) \end{array}$$

Next, the message update rule for $\alpha_{ij}$ is presented. To obtain $\alpha_{ij}$, function $R_{j}\left(\left\{x_{ij}\right\}\right)$ is minimized for two cases of $x_{ij}=1$ and $x_{ij}=0$, and the difference of their results is calculated. The resulting message update rule varies according to the relationship between *i* and *j*. If i≠j, the corresponding message is obtained as

(17) $$\begin{array}{ll} \alpha_{ij} & =\mu_{R_{j}\to x_{ij}}\left(x_{ij}=1\right)-\mu_{R_{j}\to x_{ij}}\left(x_{ia}=0\right)\\ & =\min_{X_{j}\backslash x_{ij}}R_{j}\left(x_{ij}=1,X_{j}\backslash x_{ij}\right)+\sum_{k\in N(j)\backslash i}\mu_{x_{kj}\to R_{j}}\left(x_{kj}\right)-\min_{X_{j}\backslash x_{ij}}R_{j}\left(x_{ij}=0,X_{j}\backslash x_{ij}\right)+\sum_{k\in N(j)\backslash i}\mu_{x_{kj}\to R_{j}}\left(x_{kj}\right)\\ & =\min[f\left(1,1\right)+\rho_{jj},f\left(1,2\right)+\rho_{jj}+\min_{k\neq i}\rho_{kj},f\left(1,3\right)+\rho_{jj}+\sum_{l=1}^{2}\underset{k\neq i}{\mathrm{rnk}}\left(l\right)\rho_{kj},\ldots ,\\ & \;f\left(1,N-1\right)+\rho_{jj}+\sum_{l=1}^{N-2}\underset{k\neq i}{\mathrm{rnk}}\left(l\right)\rho_{kj}]\\ & \;-\min[f\left(0,0\right),f\left(1,0\right)+\rho_{jj},f\left(1,1\right)+\rho_{jj}+\min_{k\neq i}\rho_{kj},f\left(1,2\right)+\rho_{jj}+\sum_{l=1}^{2}\underset{k\neq i}{\mathrm{rnk}}\left(l\right)\rho_{kj},\ldots ,\\ & \;f\left(1,N-2\right)+\rho_{jj}+\sum_{l=1}^{N-2}\underset{k\neq i}{\mathrm{rnk}}\left(l\right)\rho_{kj}]\\ & =\min[f\left(1,1\right),f\left(1,2\right)+\min_{k\neq i}\rho_{kj},f\left(1,3\right)+\sum_{l=1}^{2}\underset{k\neq i}{\mathrm{rnk}}\left(l\right)\rho_{kj},\ldots ,f\left(1,N-1\right)+\sum_{l=1}^{N-2}\underset{k\neq i}{\mathrm{rnk}}\left(l\right)\rho_{kj}]\\ & \;-\min[-\rho_{jj},f\left(1,0\right),f\left(1,1\right)+\min_{k\neq i}\rho_{kj},f\left(1,2\right)+\sum_{l=1}^{2}\underset{k\neq i}{\mathrm{rnk}}\left(l\right)\rho_{kj},\ldots ,f\left(1,N-2\right)+\sum_{l=1}^{N-2}\underset{k\neq i}{\mathrm{rnk}}\left(l\right)\rho_{kj}],\\ & =A_{ij}\left(1,N-1\right)-\min\left[-\rho_{jj},A_{ij}\left(0,N-1\right)\right], \end{array}$$

where rnk(l) denotes the *l*th smallest input and $A_{ij}\left(x,y\right)$ is defined with respect to message set $\left\{\rho_{kj}:k\neq i\right\}$ as

(18) $$\begin{array}{ll} A_{ij}\left(x,y\right)= & \min[f\left(1,x\right),f\left(1,x+1\right)+\min_{k\neq i}\rho_{kj},f\left(1,x+2\right)+\sum_{l=1}^{2}\underset{k\neq i}{\mathrm{rnk}}\left(l\right)\rho_{kj},\ldots \\ & f\left(1,x+y-1\right)+\sum_{l=1}^{y-1}\underset{k\neq i}{\mathrm{rnk}}\left(l\right)\rho_{kj}] \end{array}$$

On the other hand, if i=j, the message update rule can be derived in the similar way as

(19) $$\begin{array}{c} \alpha_{jj}=A_{j}\left(0,N\right) \end{array}$$

where $A_{j}\left(x,y\right)$ is defined similarly with respect to message set $\left\{\rho_{kj}:\forall k\right\}$ as

(20) $$\begin{array}{ll} A_{j}\left(x,y\right)= & \min[f\left(1,x\right),f\left(1,x+1\right)+\min_{i}\rho_{ij},f\left(1,x+2\right)+\sum_{l=1}^{2}\underset{i}{\mathrm{rnk}}\left(l\right)\rho_{ij},\ldots \\ & f\left(1,x+y-1\right)+\sum_{l=1}^{y-1}\underset{i}{\mathrm{rnk}}\left(l\right)\rho_{ij}] \end{array}$$

Since message $\rho_{ij}$ of (16) is a function of $\alpha_{ij}$ and vice versa, the resulting algorithm is obtained as two self-consistent update rules. To handle the self-consistency, the algorithm proceeds in an iterative way using the following equations:   

(21) $$\begin{array}{cc} \alpha_{ij}^{\left(t\right)}= & \left\{\begin{array}{cc} A_{ij}^{\left(t\right)}\left(1,N-1\right)-\min\left[-\rho_{jj}^{\left(t\right)},A_{ij}^{\left(t\right)}\left(0,N-1\right)\right] & \mathrm{if}\;i\neq j\\ A_{j}^{\left(t\right)}\left(0,N\right) & \mathrm{otherwise} \end{array}\right. \end{array}$$

(22) $$\begin{array}{cc} \rho_{ij}^{\left(t+1\right)}= & w_{ij}-\min_{k\in N(i)\backslash j}\left(w_{ik}+\alpha_{ik}^{\left(t\right)}\right) \end{array}$$

Note that, for physical deployment, member node *i* carries out the message calculations associated with $Q_{i}\left(\left\{x_{ij}\right\}\right)$ and $S_{ij}\left(\left\{x_{ij}\right\}\right)$ internally and combines them to prepare the outgoing message $\rho_{ij}$ to CH candidate node *j*. Thus, Then, $\alpha_{ij}$ and $\rho_{ij}$ message are sent to node *j*. Thus, only two types of messages $\alpha_{ij}$ and $\rho_{ij}$ suffice for the message-passing iteration.

### 3.3. Cluster Head Selection

Node *i* updates messages $\alpha_{ij}^{\left(t\right)}$ and $\rho_{ij}^{\left(t\right)}$ repeatedly at each iteration and determine its preferred state via $U_{i}=\min_{j\in N(i)}\left[\rho_{ij}^{\left(t\right)}+\alpha_{ij}^{\left(t\right)}\right]$. The iteration continues until the iteration count reaches the maximally allowed number $t_{max}$, or U changes below a predetermined threshold σ. On convergence, node *i* determines its CH using I by

(23) $$\begin{array}{c} I_{i}=\arg\min_{j\in N(i)}\left[\rho_{ij}^{\left(t\right)}+\alpha_{ij}^{\left(t\right)}\right] \end{array}$$

If $I_{i}=i$, node *i* becomes a CH node and forms a new cluster.

A brief consideration of the computational complexity is ensured here. At a single iteration, most of computational efforts are made in calculating messages of $\alpha_{ij}^{\left(t\right)}$, which requires as many operations as O(NlogN) for a single node in sorting input messages. Thus, it is expected that the worst case complexity for a single node amounts to $O(t_{max}N\log N)$ with the maximally allowed iteration number $t_{max}$. Since there are *N* nodes in the network, the overall computational complexity amounts to $O(t_{max}N^{2}\log N)$. The overall algorithm is presented in Algorithm 1.

**Algorithm 1** Cluster formation algorithm
For each (i,j), initialize t←0, $\rho_{ij}^{\left(t\right)}\leftarrow 0$, and $\alpha_{ij}^{\left(t\right)}\leftarrow 0$.

**repeat**

 Update $\alpha_{ij}^{\left(t\right)}$ using (21) and send to neighbors.
 Update $\rho_{ij}^{\left(t+1\right)}$ using (22) and send to neighbors.
 Increment t←t+1
**until**$|U_{i}^{\left(t+1\right)}-U_{i}^{\left(t\right)}|<\sigma$ for all *i* or $t>t_{max}$.
Node *i* chooses $I_{i}$ as its CH using (23).


## 4. Simulation Results

The simulation is conducted using standard Python 3 with NumPy and SciPy libraries. In this simulation, 100 sensor nodes are initially scattered at random in a 100 m × 100 m square region in between (0, 0) and (100, 100). Simulation parameters are set referring to [6] and summarized Table 1. We refer to the interval between consecutive cluster reformations as a single round, which amounts to 7 days. The predetermined cluster number required by the existing algorithms for comparison is set to K=5 [6]. Results are averaged over 100 random drops of wireless sensors. The lifetime of the WSN is calculated by the number of live nodes, which is evaluated at each round that consists of the set-up phase and the steady-state phase. The network lifetime and total amount of transferred data are measured by evaluating the number of rounds until the death of the last node.

**Figure 4:**
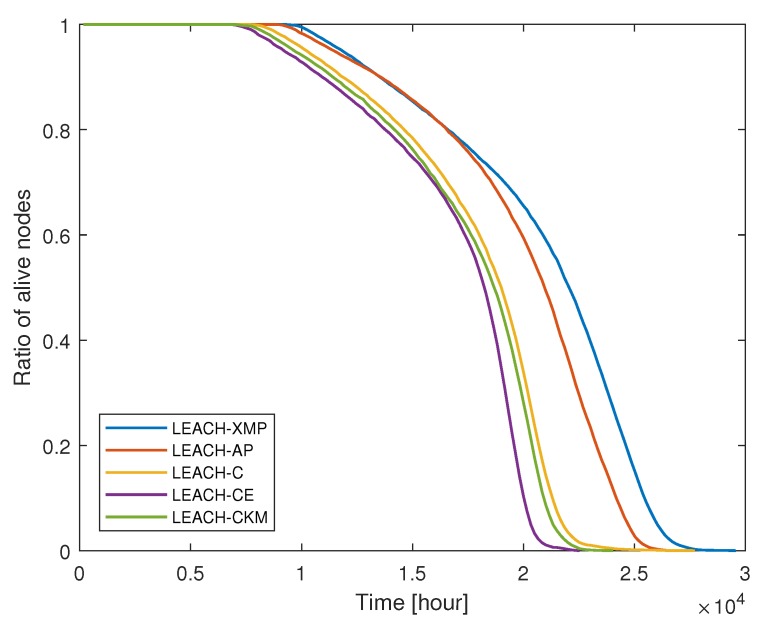
Ratio of live nodes versus time.

**Figure 5:**
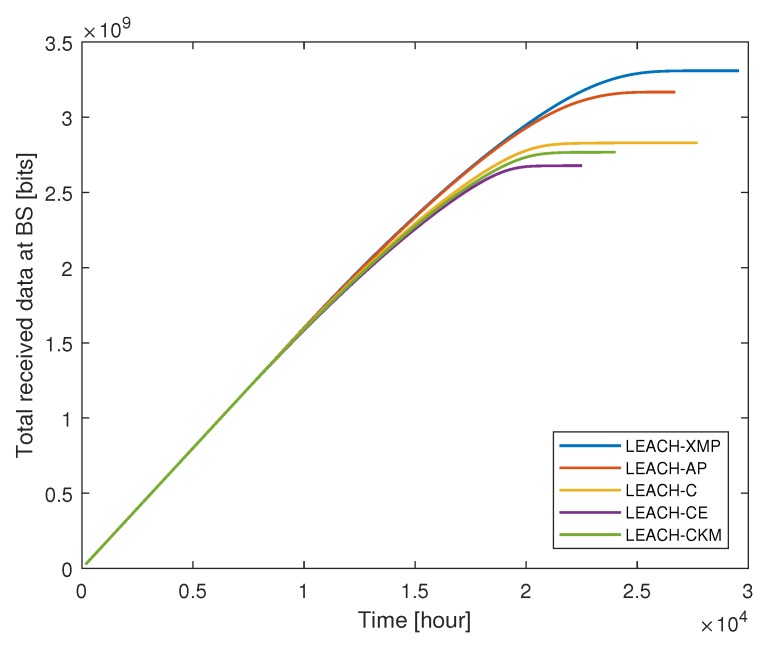
Total amount of data received at the base station (BS) versus time.

We compare the performance of the proposed LEACH-XMP with existing LEACH-based protocols including LEACH-C, LEACH-CE, LEACH-CKM, and LEACH-AP. Since the existing techniques assume that the collected data can be perfectly compressed at CH nodes, i.e., λ=1 for fair comparison, which is the special case of the proposed LEACH-XMP. Figure 4 compares the ratio of live nodes over time, while Figure 5 compares the total amount of data received at the BS over time in case A where the BS is located at (50, 175). As time elapses, the number of live nodes decreases. LEACH-XMP provides 18.7%, 28.5%, 21.5%, and 5.33% increase in the network lifetime as compared to LEACH-C, LEACH-CE, LEACH-CKM, and LEACH-AP, respectively. Accordingly, LEACH-XMP transfers the largest amount of sensing data to the BS among the LEACH variations.

Table 2 compares the computational complexity of LEACH-XMP and existing LEACH variations. In LEACH-C and LEACH-CE, the cluster formation is fulfilled by a simulated annealing algorithm [6]. At each iteration of the simulated annealing algorithm, the roles of the partial set of nodes are interchanged to improve a temporal solution, and the resulting computational complexity per iteration is low, i.e., O(tKN). However, a simulated annealing algorithm typically requires at least thousands of iterations for convergence, *t*. Specifically, we set t=60,000 in our simulations considering its slow convergence. On the other hand, our simulations results show that LEACH-CKM requires relatively small number of iterations, e.g., less than 10 iterations in most cases. In general, the performance of the *K*-medians algorithm adopted in LEACH-CKM is strongly affected by the initial choice of the value of *K* and CH nodes. Thus, the algorithm requires at least dozens of runs with different initial points to find more improved solutions. Since our simulations set the total number of runs for LEACH-CKM to 30, the overall computational complexity of LEACH-CKM increases significantly. At each iteration of message-passing, LEACH-AP requires $N^{2}$ operations all over the network, while LEACH-XMP requires $N^{2}\log N$ operations since LEACH-XMP involves the sorting operation in $\alpha_{ij}$ message calculation, which is slight increase in complexity. Note that LEACH-XMP efficiently finds a near optimal solution without multiple runs of different initialization setups and rapidly converges within dozens of iterations as will be confirmed in the following simulations results. Overall, LEACH-XMP has comparable complexity over other LEACH variations.

**Figure 6:**
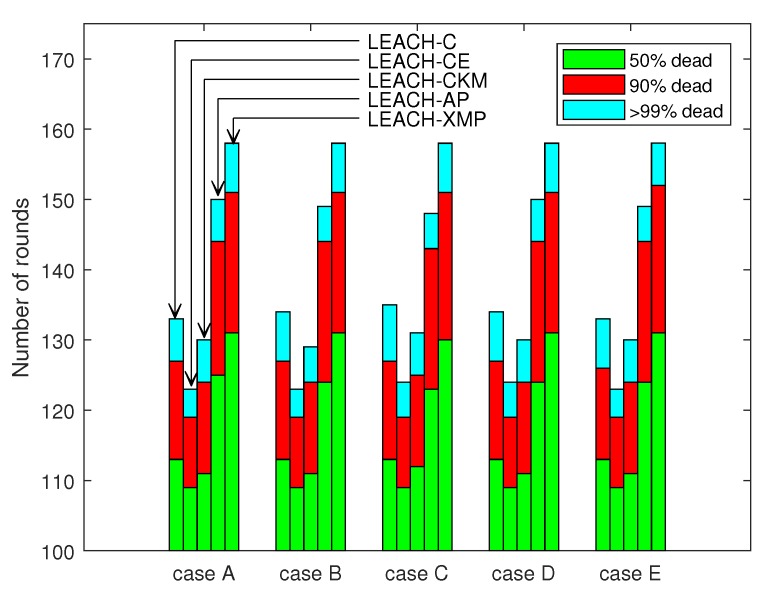
Comparison of network lifetime for various configurations.

Figure 6 shows the impact of the BS position and energy distribution. Five simulation scenarios A-E are considered as listed in Table 3. Five columns of each case correspond to LEACH-C, LEACH-CE, LEACH-CKM, LEACH-AP, and LEACH-XMP, respectively. Three stacked bars in each column are associated with the times elapsed until 50%, 90%, and 99% of the nodes are out of operation, respectively. In all cases, LEACH-XMP outperforms in terms of the lifetime of network, irrespective of the position of the BS. In particular, the 50%-node-survival time is enhanced considerably for the proposed algorithm. This shows that LEACH-XMP sophisticatedly optimizes nonlinear energy consumption which has not been properly handled previously.

**Figure 7:**
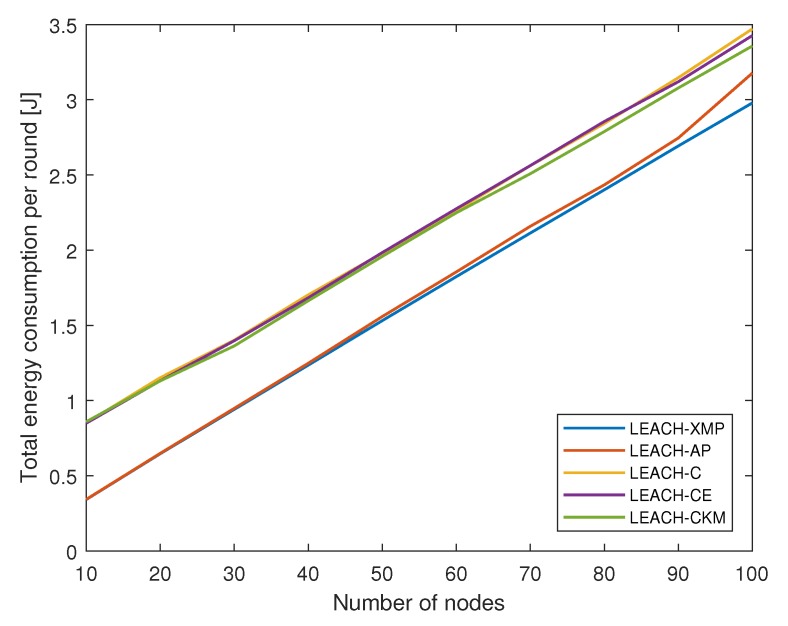
Total energy consumption per round versus number of sensor nodes.

Figure 7 shows how the total energy consumption of the network on a single round varies with the number of sensor nodes. As the network grows, the total energy consumption increases gradually. As compared to existing LEACH variations, message-passing-based techniques, i.e., LEACH-AP and LEACH-XMP, provide energy-efficient solutions. Furthermore, the performance improvement of the proposed LEACH-XMP over LEACH-AP becomes noticeable as the network size increases, in that the proposed LEACH-XMP finds a more energy-efficient solution than LEACH-AP from the consideration of the generalized energy consumption model for CH nodes.

**Figure 8:**
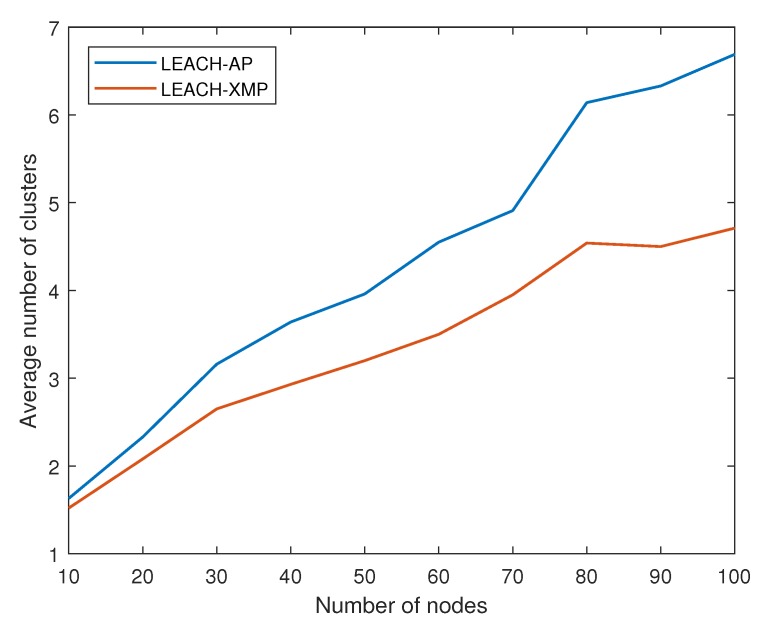
Number of clusters versus number of sensor nodes.

Figure 8 shows how the average number of clusters changes depending on network size. As the network grows, the average number of clusters increases in both LEACH-AP and LEACH-XMP. However, the proposed algorithm maintains a small number of clusters comparing to LEACH-AP. LEACH-AP uses a simplified energy consumption model by ignoring non-linear terms. Thus, it normally underestimates the energy consumption of CH nodes, creating more number of clusters. By adopting a realistic energy consumption model, the proposed LEACH-XMP can identify an efficient solution for dynamic cluster sizes in large-scale networks. This explains the total energy consumption performance in Figure 7.

**Figure 9:**
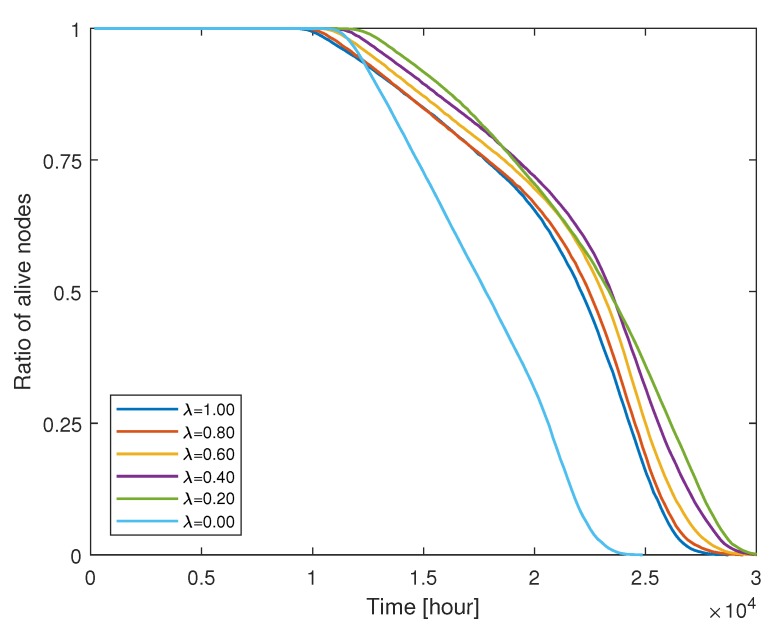
Measurements of live nodes per time for various λ configurations.

We now consider a general case of λ,(λ<1), i.e., non-ideal compression at CH nodes. According to (4) and Figure 2, the compressed data size to be delivered from CH nodes to the BS varies depending on λ and cluster size. The compressed data size dominates the energy consumption of CH nodes and the overall cluster formation. Figure 9 shows how the fraction of live nodes reduces as time elapses in the simulation. In all regimes of the λ value, the remaining energy of nodes gradually become depleted and the nodes are excluded from the network. If the cluster formation is identical, a low value of λ leads to the increase in energy consumption of CH as expected from (6). However, the lifetime of the network still extends even in a low λ regime. The clustered results from LEACH-XMP offset the increment of $b_{n}$. CH nodes are relieved from the burden to deliver a large amount of the data compressed with a very low rate and saves the remaining energy for survival. Thus, LEACH-XMP outperforms in a general case of λ<1 as compared to previously proposed protocols which fail to compensate the effect by lower λ. If λ=0, $b_{n}$ increases quickly as Figure 2 and the cluster formation alone cannot completely compensate for this effect.

**Figure 10:**
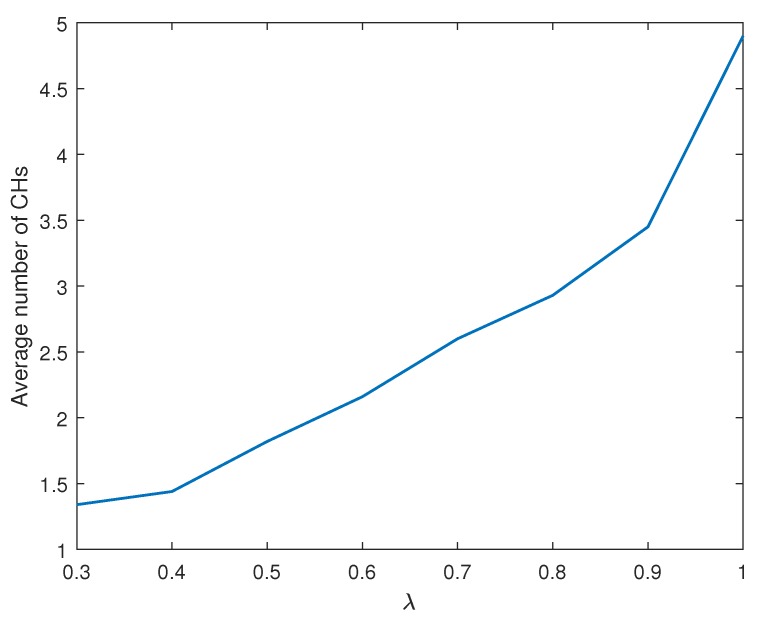
Average number of cluster versus λ.

Figure 10 illustrates how the average number of clusters varies with the rate of the data compression. The average number of clusters gradually increases with λ. With small λ, the amount of compressed data at CH nodes is large, causing the energy consumption burden to the CH nodes. Accordingly, the network becomes energy-efficient when it maintains a lower number of clusters. By contrast, the amount of the compressed data at CH nodes is small with a large λ. This implies a lower energy consumption burden to the CH nodes, thereby populating more clusters in the network.

**Figure 11:**
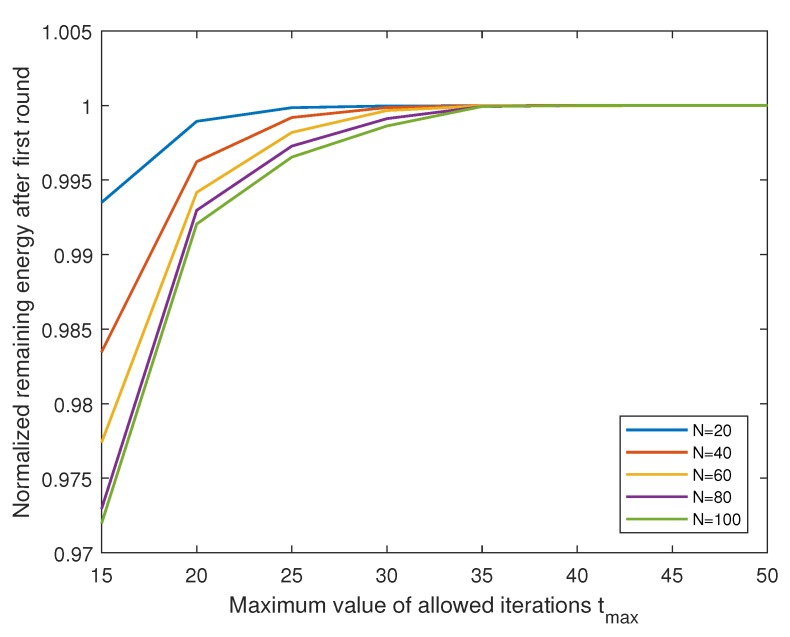
Convergence of LEACH-eXtended Message-Passing (LEACH-XMP).

Figure 11 shows how fast the proposed LEACH-XMP converges in various network sizes. In all ranges of the network size, the proposed LEACH-XMP converges at the latest within 35 iterations. In general, a larger network size requires slightly longer convergence time. However, the performance difference becomes indistinguishable after 10 to 20 iterations. With the low signaling overhead, the proposed LEACH-XMP is beneficial for practical implementations.

## 5. Conclusions

This work develops LEACH-XMP, a LEACH improvement via an enhanced message-passing framework, which maximizes the remaining energy and thus the lifetime of the WSN by considering realistic LEACH-based model. To suggest an efficient solution for this model, nonlinear energy consumption for CH nodes is included in the network energy consumption minimization, enabling an advanced energy-efficient cluster construction. By utilizing the proposed protocol, individual nodes can adaptively form an energy-efficient cluster network regardless of the compression rate of the data. The proposed LEACH-XMP consistently outperforms existing LEACH techniques without absolute positioning of each sensor, predetermination of the number of clusters, and centralized control of the BS. Such an advantage of LEACH-XMP originates from the fact that LEACH-XMP sophisticatedly optimizes nonlinear realistic energy consumption accommodating energy-efficient compressed representation of the sensing data, which has not been properly handled in existing LEACH techniques.

## Figures and Tables

**Table 1:** Simulation Parameters.

Description	Value
Radio circuitry energy dissipation, $E_{elec}$	50 nJ/bit
Energy dissipation of amplifier in free-space, $\epsilon_{fs}$	10 pJ/bit/m${}^{2}$
Energy dissipation of amplifier in multipath, $\epsilon_{mp}$	0.0013 pJ/bit/m${}^{4}$
Energy consumption for data aggregation, $E_{da}$	5 nJ/bit/signal
Initial energy of each sensors, $E_{0}$	2 J
Initial number of sensors	100
Reporting interval	1 h
Cluster reformation interval	7 days
Packet size (/node/report)	200 bytes
Maximum value of allowed iterations, $t_{max}$	50
Stopping threshold, σ	0.001

**Table 2:** Computational Complexity.

Protocol	Complexity
LEACH-C	O(tKN)
LEACH-CE	O(tKN)
LEACH-CKM	O(tKN)
LEACH-AP	$O(tN^{2})$
LEACH-XMP	$O(tN^{2}\log N)$

**Table 3:** Simulation Scenarios.

Case	Scenario Description
A	BS = (50, 175), $E_{0}$ = 2 J
B	BS = (50, 125), $E_{0}$ = 2 J
C	BS = (50, 150), $E_{0}$ = 2 J
D	BS = (50, 200), $E_{0}$ = 2 J
E	BS = (50, 175), $E_{0}$ = unif(1,3) J

## Abbreviations

The following abbreviations are used in this manuscript:

LEACH	Low-Energy Adaptive Clustering Hierarchy
WSN	Wireless Sensor Network
PEGASIS	Power-Efficient Gathering in Sensor Information Systems
HEED	Hybrid Energy-Efficient Distributed clustering
EEUC	Energy-Efficient Uneven Clustering
LEACH-XMP	LEACH-eXtended Message-Passing
LEACH-AP	LEACH-Affinity Propagation
LEACH-C	LEACH-Centralized
LEACH-CE	LEACH-Centralized Efficient
LEACH-CKM	LEACH-C with K-Means and Minimum Transmission Energy routing protocol
CH	Cluster Head
BS	Base Station
